# Identification and drug resistance profiling of *Mycobacterium orygis* from an African elephant using whole-genome sequencing

**DOI:** 10.1128/spectrum.03586-25

**Published:** 2026-05-07

**Authors:** Hassan Ghayas, Javaria Ashraf, Noureen Saeed, Sadaf Zaka, Samreen Shafiq, Joveria Farooqi, Kauser Jabeen, Abida Rais, Aamir Ismail Rizvi, Ghulam Mustafa, Samreen Ashraf Qureshi, Yusra Askari, Naseem Salahuddin, Egon A. Ozer, Rumina Hasan

**Affiliations:** 1 Department of Pathology and Laboratory Medicine, The Aga Khan University 9615, Karachi, Pakistan; 2 Karachi Zoo, Karachi Metropolitan Corporation, Karachi, Pakistan; 3 Department of Pathology, University of Veterinary and Animal Sciences 66920, Lahore, Pakistan; 4 TB Control Program, Health Department, Government of Sindh 127502 , Karachi, Pakistan; 5 Ministry of Local Government (Zoo Safari), Government of Sindh 127502 , Karachi, Pakistan; 6 Indus Hospital, Karachi, Pakistan; 7 Feinberg School of Medicine, Northwestern University 12244, Chicago, Illinois, USA; 8 London School of Hygiene and Tropical Medicine 4906, London, United Kingdom; City of Hope, Duarte, California, USA

**Keywords:** *Mycobacterium orygis*, African elephant, whole-genome sequencing, resistance profiling, isoniazid resistant

## Abstract

Tuberculosis (TB) caused by members of the *Mycobacterium tuberculosis* complex (MTBC) has been increasingly reported in wildlife and captive animals, posing zoonotic risks. Accurate diagnosis and speciation are essential to trace the infection source and to tailor therapy accordingly. Current diagnostic tools, however, have limitations in speciating MTBC strains. In this study, we investigated a lung biopsy sample of a deceased adult female elephant (*Loxodonta africana*) for mycobacterial infection. Mycobacterial Growth Indicator Tube culture was positive, and initial immunochromatographic testing (BD MPT64) confirmed the isolate as a member of the MTBC. To further confirm species identity and assess drug resistance, whole-genome sequencing was performed. Species was identified using Genome Detective and KmerFinder. Species identification and lineage analysis consistently classified the isolate as *Mycobacterium orygis* (lineage La3)*,* a rare and often overlooked zoonotic member of the MTBC. Antimicrobial resistance profiling through TB-Profiler and PhyResSE identified resistance to isoniazid, attributed to the presence of the katG p.Ser315Thr mutation. No resistance-associated mutations were detected for rifampicin, levofloxacin, or pyrazinamide. These findings were consistent with phenotypic drug susceptibility testing. The detection of *M. orygis* in a captive elephant suggests that the infection was of zoonotic origin and underscores its potential for spreading disease, and thus the need for integrated surveillance in animal reservoirs.

**IMPORTANCE:**

This study reports the first case of *Mycobacterium orygis* infection in an African elephant in captivity. Currently, the methods for differentiation of *Mycobacterium tuberculosis* complex (MTBC) subspecies have limitations. This case highlights the importance of precise species identification and drug resistance profiling of MTBC organisms and the use of whole-genome sequencing in diagnosis. Additionally, our findings underline the need for surveillance of mycobacteria and other potential pathogens in captive animals and in animal caregivers to reduce the potential for zoonotic disease transmission.

## OBSERVATION

Tuberculosis (TB) remains a major health problem affecting both humans and animals worldwide (1). While TB is primarily caused by *Mycobacterium tuberculosis*, other members of *Mycobacterium tuberculosis* complex (MTBC) also contribute to the disease in a variety of hosts (2). Several MTBC species cause TB in domestic cattle, wild, and captive animals and have the potential for spreading zoonotic infection (3, 4). Among these, *Mycobacterium orygis* (previously known as *Oryx bacilli*) has increasingly been reported from South Asia, where it is considered endemic, with India reporting the highest prevalence (5, 6). *M. orygis* has only been identified in slaughtered cattle from Pakistan (7), whereas its presence in humans in Pakistan has not been documented. However, human cases of *M. orygis* infection have been described in India, Australia, the USA, and Canada (8–11). Cross-species transmission of *M. orygis*, such as transmission from an immigrant from South Asia to a dairy cow in New Zealand (12), as well as the emergence of infection in humans, underscores its zoonotic potential.

We report *M. orygis* infection in a deceased adult female African elephant (*Loxodonta africana*) that had been housed at Karachi Safari Park for 15 years. A postmortem lung biopsy specimen was collected for mycobacterial culture following clinical suspicion of tuberculosis. GeneXpert MTB/RIF (Cepheid, Sunnyvale, CA, USA) assay was performed to detect the MTBC DNA and rifampicin resistance in the sample. Following decontamination, the sample was inoculated into BACTEC 960 Mycobacterial Growth Indicator Tube (MGIT) system (Becton Dickinson Diagnostic Instruments Systems, Sparks, MD, USA). The MGIT system indicated mycobacterial growth, which was further confirmed as a member of MTBC using BD-MPT64 immunochromatographic testing kit (Becton Dickinson Diagnostic Instruments Systems, Sparks, MD, USA). Given the inability of the immunochromatographic assay to differentiate the MTBC subspecies, whole-genome sequencing (WGS) of the isolate was performed. DNA was extracted using the InstaGene Matrix kit (Bio-Rad Laboratories, Hercules, CA, USA), as described previously (13). A DNA library for sequencing was prepared using the Nextera XT DNA Library Preparation Kit (Illumina, USA) and indexed with Nextera XT Index Kit (Illumina, USA) according to the manufacturer’s protocol. Sequencing was performed on the Illumina MiniSeq platform using the MiniSeq Mid-output kit to generate 2 × 151 bp paired-end reads.

The whole-genome sequence was analyzed with vSNP3 v.3.30 pipeline (https://github.com/USDA-VS/vSNP3) to align reads and assign MTBC species (14). Identity of the organism was further determined by KmerFinder v3.0.2 (15) and Genome Detective webtool v2.23.0 (16). Analysis of WGS data using the vSNP3 pipeline and KmerFinder identified the isolate as *M. orygis*, a member of MTBC. Genome detective web tool further confirmed the species identification as *M. orygis* with 91.2% of genome coverage and 65.7× depth of coverage against *M. orygis* reference genome (NZ_CP063804). Additionally, TB-Profiler v4.4.2 classified the isolate within the La3 phylogenetic lineage, representing *M. orygis*, thus providing further support for species assignment.

Drug susceptibility testing for isoniazid, rifampicin, pyrazinamide, and levofloxacin was performed using BACTEC MGIT 960 SIRE kit (Becton Dickinson Diagnostic Instruments Systems, Sparks, MD, USA). Phenotypic susceptibility testing identified that the isolate was resistant to isoniazid but susceptible to rifampicin, levofloxacin, and pyrazinamide (Table 1). TB-Profiler pipeline v4.4.2 (17, 18) and PhyResSe v1.0 tool (19) were used to predict the drug resistance using the whole-genome sequence. *In silico* identification of resistance-associated mutations using TB-Profiler and PhyResSe tool detected the KatG p.Ser315Thr mutation responsible for isoniazid resistance (Table 1). No resistance-associated mutations were found in the *rpoB*, *gyrA*/*gyrB*, *pncA*, and *embB* genes associated with resistance to rifampicin, fluoroquinolones (levofloxacin), pyrazinamide, and ethambutol, respectively; hence, the isolate was classified as Hr-TB (isoniazid-resistant tuberculosis) drug resistance type. This high concordance between phenotypic and genotypic results strengthens confidence in the resistance profiling using WGS and highlights its importance in resistance prediction in MTBC isolates.

**TABLE 1:** Antimicrobial resistance profile of *Mycobacterium orygis* isolate

Antibiotics	Phenotypic results	Resistance-associated mutation	Inferred genotypic results
Isoniazid	Resistance	KatG p.Ser315Thr	Resistance
Rifampicin	Sensitive	–^*a*^	Sensitive
Levofloxacin	Sensitive	–	Sensitive
Pyrazinamide	Sensitive	–	Sensitive
Ethambutol	Not determined	–	Sensitive

^*a*^ – indicates that resistance-associated mutations were not found for the respective drug.

**Fig 1:**
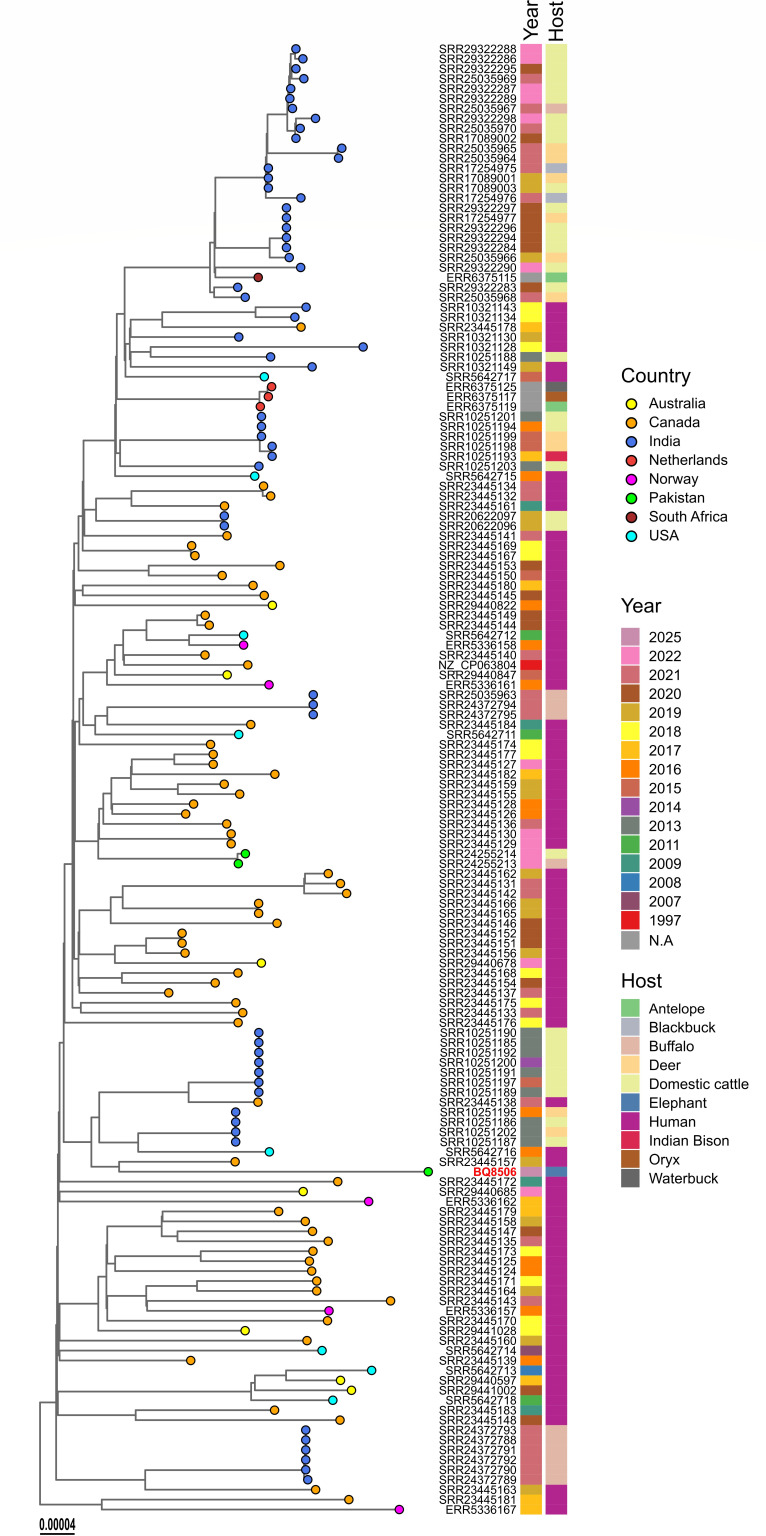
Phylogenetic relationship of *Mycobacterium orygis* isolates. Maximum-likelihood phylogenetic tree constructed from core genome SNPs. Tree was rooted on the H37Rv (GenBank accession: NC_000962.3) genome as an outgroup, and the outgroup tip was dropped from the final tree. Tips are colored by the country of isolation. The isolate sequenced in this study (BQ8506) is indicated by a red taxon label.

To place the elephant isolate in a global context, a whole-genome phylogenetic analysis was performed using publicly available *M. orygis* sequences from NCBI-SRA database. Reads were mapped against the *M. orygis* reference genome (GenBank accession: NZ_CP063804.1) using the snippy pipeline v4.6.0 (https://github.com/tseemann/snippy). Whole-genome multi-alignment was generated using snippy-core, and variable sites were identified using snp-sites (v2.5.1) (20) to generate the core genome single-nucleotide polymorphism (SNP) alignment. A maximum likelihood phylogenetic tree was constructed using IQ-TREE (v2.4.0) (21) with 1,000 bootstrap iterations using ultrafast bootstrap approximation (22). Tree was visualized and annotated in R v4.4.2 using ggtree package v3.10.1 and rooted on outgroup using APE package v5.8.1. A whole-genome phylogenetic tree based on SNPs revealed that our study isolate clustered with *M. orygis* clade and was closely related to a Canadian isolate collected from a human with tuberculosis, with the distance of 110 SNPs (Fig. 1). Previously sequenced Pakistani isolates cluster distantly from our isolate by 138 SNPs which suggests that the isolate from this study represents a distinct lineage with no epidemiological linkage. Notably, the phylogenetic analysis revealed that isolates from humans, wildlife, and livestock were distributed across the phylogeny, with no evidence of host-specific clustering or distinct lineages. This interspersed distribution of isolates in the phylogenetic tree from a variety of hosts suggests that *M. orygis* is capable of infecting multiple host species. Evidence of cross-species transmission has been reported previously, including a case in New Zealand involving transmission from a human to dairy cattle, indicating that *M. orygis* can cross host boundaries under certain conditions (12). Marcos et al. also reported eight cases of *M. orygis* infection in New York in South Asian emigrants with no evidence of epidemiological clustering (9). In another study, transmission of an indistinguishable strain of *M. tuberculosis* from an Asian elephant to a chimpanzee in an Australian zoo has been reported (3). Such inter-species transmission underscores the One Health implication of *M. orygis* where animals and humans are both at risk of infection.

*M. orygis* was previously identified in slaughtered cattle and buffalo with TB-like lesions from Lahore, Pakistan (7), suggesting its presence in a livestock reservoir in this region. The identification of *M. orygis* in a captive elephant expands the known host range of this MTBC species in this region. *M. orygis* infections in humans have also been reported from the USA and Canada, with most of the patients originating from India, Bangladesh, Nepal, or Pakistan (9, 11). However, to date, *M. orygis* infection has not been reported in humans in Pakistan. This may be due to a lack of diagnostic tools and techniques to speciate MTBC infections in clinical settings and highlights the limitations of routine diagnostic methods for species-level identification. In settings where MTBC infections are presumed to be caused by *M. tuberculosis* or *M. bovis*, infections due to *M. orygis* may remain undetected or misclassified. This has important implications for surveillance, treatment, and control strategies, particularly in regions with close human-animal interfaces. The detection of isoniazid resistance in this isolate further emphasizes the need for rapid and accurate resistance profiling, as drug-resistant MTBC strains circulating in animal reservoirs may complicate TB control efforts in both veterinary and human health sectors. A recent report had described predominantly isoniazid-susceptible *M. orygis* isolates from wildlife in India. However, isoniazid-resistant MTBC has also been identified in fecal samples of blackbuck in the same animal enclosures, although species-level identification was not determined, suggesting the potential for emergence of resistance within this lineage (23).

In summary, identification of *M. orygis* in an elephant highlights the importance of strain identification using WGS, as well as the need for the surveillance of *M. orygis* and other potential pathogens in captive animals. Identification of pathogens is important to trace the source of the organism and implement preventive measures to control the cross-transmission of the pathogen. This study also demonstrates the need for robust molecular diagnostic assays for the differentiation of MTBC species in diagnostic labs. Moreover, rapid profiling of drug resistance in mycobacteria using WGS is essential for effective treatment and disease control. Collectively, in the context of a One Health approach, our findings emphasize the importance of regular screening of animal workers and caregivers to reduce the risk of zoonotic transmission of disease in captive and high-risk environments.

## Data Availability

The whole-genome sequence of *M. orygis* was submitted to the NCBI Sequence Read Archive (SRA) database under BioProject accession number PRJNA1353862.
